# The combination of salvianolic acid A with latamoxef completely protects mice against lethal pneumonia caused by methicillin-resistant *Staphylococcus aureus*

**DOI:** 10.1080/22221751.2020.1711817

**Published:** 2020-01-23

**Authors:** Dan Mu, Yongxin Luan, Lin Wang, Zeyuan Gao, Panpan Yang, Shisong Jing, Yanling Wang, Hua Xiang, Tiedong Wang, Dacheng Wang

**Affiliations:** a College of Animal Science, Jilin University, Changchun, People’s Republic of China; b Department of Neurosurgery, First Hospital of Jilin University, Jilin University, Changchun, People’s Republic of China; c Key Laboratory of Zoonosis Research, Ministry of Education, College of Veterinary Medicine, Jilin University, Changchun, People’s Republic of China; d Department of Pharmacology, College of Basic Medical Science, Jilin University, Changchun, People’s Republic of China; e Qingdao Vland biological Limited co., LTD, Qingdao, People’s Republic of China; f College of Animal Science and Technology, Jilin Agricultural University, Changchun, People’s Republic of China

**Keywords:** *Staphylococcus aureus*, sortase A, salvianolic acid A, pneumonia, inhibitor

## Abstract

*Staphylococcus aureus* (*S. aureus*), especially methicillin-resistant *Staphylococcus aureus* (MRSA), is a major cause of pneumonia, resulting in severe morbidity and mortality in adults and children. Sortase A (SrtA), which mediates the anchoring of cell surface proteins in the cell wall, is an important virulence factor of *S. aureus*. Here, we found that salvianolic acid A (Sal A), which is a natural product that does not affect the growth of *S. aureus*, could inhibit SrtA activity (IC_50_ = 5.75 μg/ml) and repress the adhesion of bacteria to fibrinogen, the anchoring of protein A to cell wall, the biofilm formation, and the ability of *S. aureus* to invade A549 cells. Furthermore, *in vivo* studies demonstrated that Sal A treatment reduced inflammation and protected mice against lethal pneumonia caused by MRSA. More significantly, full protection (a survival rate of 100%) was achieved when Sal A was administered in combination with latamoxef. Together, these results indicate that Sal A could be developed into a promising therapeutic drug to combat MRSA infections while limiting resistance development.

## Introduction

*Staphylococcus aureus* (*S. aureus*) is a widespread bacterial pathogen that is capable of causing many diseases with varying severity, such as skin and soft-tissue infections, pneumonia, sepsis, and endocarditis [1,2]. The emergence and prevalence of methicillin-resistant *S. aureus* and vancomycin-resistant *S. aureus* pose serious threats to public health [3] and has indicated a need for the development of innovative anti-infective approaches to control infections caused by these pathogens.

The pathogenesis of *S. aureus* is regulated by a large repertoire of virulence proteins, including surface-associated proteins and secreted toxins [4]. These virulence proteins are involved in various stages of the infectious cycle by mediating the attachment of *S. aureus* to host cells or extracellular matrix components, inhibiting complement activity, reducing antibody function, damaging host cells, and evading immune elimination [5,6]. Therefore, the therapeutic targeting of virulence factors can reduce the pathogenicity of bacteria and help the host immune system clear pathogens and may serve as a promising approach to control *S. aureus* infections [7].

Many cell wall-anchored proteins are important virulence factors of *S. aureus* due to their roles in the colonization and invasion of host tissues [8]*.* These proteins are anchored to the bacterial surface by a class of transpeptidases known as sortase A (SrtA) in *S. aureus* [9]. The *S. aureus* SrtA mutant strain*,* lacking all cell wall-anchored proteins, has shown markedly reduced virulence in various mouse infection models [10,11]. Therefore, blocking the display of cell wall-anchored proteins by inhibiting the activity of SrtA using inhibitors can reduce bacterial virulence and promote bacterial clearance by the host immune system [12]. As an enzyme on the bacterial membrane, SrtA is more susceptible to targeting by inhibitors than intracellular bacterial targets. Furthermore, because SrtA is not an essential component of bacterial growth and proliferation, the inhibition of SrtA will neither lead to bacterial resistance nor affect bacteria in the normal flora of the host. Therefore, SrtA is a particularly promising target for combating *S. aureus* infections [13].

To date, several classes of SrtA inhibitors have been investigated, including compounds from synthetic product libraries, well-designed peptidomimetics and natural products [14]. Among them, natural products, especially small molecule drugs from traditional Chinese herbal medicines, have received great attention as a new source of anti-virulence drugs. Here, we revealed that the natural compound salvianolic acid A (Sal A) is an effective inhibitor of SrtA. Sal A is a water-soluble phenolic compound contained in the dried roots and rhizomes of *Salvia miltiorrhiza Bunge*, and it has significant anti-oxidation, myocardial ischaemia protection, anti-thrombosis, neuroprotective, and anti-fibrosis effects and has been used to prevent and treat diabetes and address complications, among other pharmacological activities [15–17].

In the present work, we systematically studied the inhibitory effect of Sal A on SrtA activity and further investigated its mechanism of inhibition. In addition, we evaluated the therapeutic effects of Sal A on MRSA-induced lethal pneumonia in a mouse model. We showed that Sal A is a promising anti-virulence agent and can be used to control MRSA infections.

## Materials and methods

### Bacterial strains and chemicals

The *S. aureus* strain used throughout this study was the USA300 strain LAC, which was obtained from the American Type Culture Collection (Rockville, MD), and the *srtA* deletion mutant (Δ*srtA* strain), which was a gift from Dr. Xuming Deng [18]. The peptide substrate Abz-LPATG-Dap(Dnp)-NH_2_ (Abz:ortho-aminoben-zoic acid; Dnp:2,4-dinitrophenyl) was manufactured by GL Biochem (Shanghai, China). The compound Sal A was purchased from the Chengdu Ruifensi Biotech Company (Chengdu, China).

### Cloning, expression, and purification of SrtA

The gene encoding SrtA (lacking the N-terminal transmembrane domain (N_1–59_)) was amplified from *S. aureus* genomic DNA using the primers *srtA*-F and *srtA*-R by PCR. The resulting amplified fragment was digested with BamHI and NdeI and ligated into the same sites of pET28a, yielding pET28a-SrtA. Site-directed mutagenesis to introduce the substitutions A104L, A92L, I182A and R197A into SrtA was performed using the QuickChange site-directed mutagenesis kit (TransGen Biotech). All primers are listed in Table 1. The expression and purification of recombinant 6 × His-tagged SrtA and the mutant SrtA were carried out as previously described [19].

**Table 1.** Primers used in this study.

Primer name	Sequences (5′-3′)
*srtA*-F	GGGAATTCCATATGCAAGCTAAACCTCAAATTCCG
*srtA*-R	CGCGGATCCTTATTTGACTTCTGTAGCTACAAAGA
A92L-*srtA*-F	GACCAAAAACACCTGAACAATTAAA
A92L-*srtA*-R	CTGGATATACTGGTTCTTTAATATCAGC
A104L-*srtA*-F	CTTTAAAGAAGAAAATGAATCACTA
A104L-*srtA*-R	CTTACACCTCTATTTAATTGTTCAG
I182A-*srtA*-F	AACATTAGCTACTTGTGATGATTAC
I182A-*srtA*-R	AATTGTTTATCTTTACCTTTTTGTTCA
R197A-*srtA*-F	GGAAAAAGCTAAAATCTTTGTAGCT
R197A-*srtA*-R	CAAACGCCTGTCTTTTCATTG

### SrtA activity assay

The SrtA activity assay was performed by using FRET as described previously [20,21]. Briefly, a mixture of 4 μM recombinant SrtA and different concentrations of Sal A in a 300 μl reaction volume (50 mM Tris·HCl, 150 mM NaCl and 5 mM CaCl_2_, pH 7.5) was incubated for 1 h at 37°C. Then, 10 μM substrate peptide was added and incubated for an additional hour. Wells containing all constituents except SrtA served as blank controls. The fluorescence was measured with a microplate reader at λ_ex_ = 350 nm and λ_em_ = 495 nm.

### Reversible inhibition assay of SrtA

To evaluate the reversible inhibition by Sal A of SrtA, 100 μl of purified SrtA was incubated with Sal A at a final concentration equal to 10-fold the IC_50_ for 1 h at 37°C. Then, 9.9 ml reaction buffer was added. Subsequently, 10 μl of the substrate peptide was added to the 190 μl diluted mixture at a final concentration of 10 μM. The fluorescence intensity was measured using a microplate reader at λ_ex_ = 350 nm and λ_em_ = 495 nm. Triplicate measurements were obtained for each data point, and the data are reported as the mean ± SEM.

### Analysis of the anti-*S. aureus* activity of Sal A

The minimum inhibitory concentration (MIC) of Sal A was determined by broth microdilution according to the NCCLS guidelines. Briefly, overnight cultures of *S. aureus* were diluted 1:100 with fresh Brain Heart Infusion (BHI) medium supplemented with or without 256 μg/ml Sal A and grown at 37°C with shaking. Absorbance readings were obtained at OD_600_ at different time intervals.

### Fibrinogen binding assay

Overnight cultures of *S. aureus* were subcultured into fresh medium with or without Sal A and then grown until the OD_600_ reached 0.5. Then, 100 μl of the bacterial culture was transferred into each well of a polystyrene 96-well plate coated with 20 μg/ml bovine fibrinogen and incubated for 2 h at 37°C. The cell suspension was discarded, and the wells were rinsed twice with PBS. Then, 25% formaldehyde was added and incubated for 30 min, and the plate was washed three times and stained with crystal violet dye (12.5 g/l). The binding of *S. aureus* to fibrinogen was quantitated as described previously [11]. The data are presented as the mean ± SEM from three separate experiments.

### Biofilm formation assay

Overnight cultures of *S. aureus* were diluted 1:100 in BHI media with or without Sal A to an OD_600_ of 0.6. Then, 5 µl of the bacterial culture was added to 195 µl BHI media with or without Sal A in a 96-well plate. The plate was incubated at 37°C for 18 h to form a biofilm, which was then quantitated as described previously [22].

### S. *aureus* protein A (SpA)-related fluorescence analysis

An overnight culture of *S. aureus* was diluted 1:100 in BHI broth with or without Sal A and grown until the OD_600_ reached 1.0. After centrifugation at 2,500 × g for 10 min, the bacterial pellets were fixed with 4% formaldehyde solution for 30 min. After washing three times with PBS, 5% BSA was added for blocking, and the bacteria were incubated for 20 min. Then, the bacteria were rinsed with PBS and resuspended in PBS containing FITC-labelled rabbit anti-goat-IgG (Sigma) at a dilution of 1:50 and incubated at 37°C for 3 h. After washing three times with PBS, the bacteria were placed onto poly-lysine-coated glass slides, and the SpA on the cell surface was visualized by confocal microscopy (Zeiss, LSM 880).

### Flow cytometry

To quantify the effect of the Sal A on the anchoring of SpA, the FITC-IgG-stained *S. aureus* was measured by flow cytometry. *S. aureus* strains were grown overnight and then were diluted 1:100 in BHI broth with or without Sal A and grown until the OD_600_ reached 1.0. Then, bacteria were harvested by centrifugation at 2,500 × g for 10 min. The collected pellets were washed once with PBS and were incubated in PBS solution containing 0.2% BSA (wt/vol) for 10 min. Afterward, bacteria were washed thrice and then suspended in PBS containing FITC-labelled rabbit anti-goat-IgG at a dilution of 1:50 and incubated at 37°C in the dark for 60 min. After a thorough washing, bacteria were fixed with formaldehyde (2%, vol/vol) for 10 min at room temperature. Fluorescence signal was detected on a FACSCanto flow cytometer (Becton Dickinson, USA) and analyzed using FACSDiva software.

### Invasion assay

The assay of the internalization of *S. aureus* into A549 cells was performed as described [23]. Briefly, 3 × 10^5^ A549 cells were plated in each well of a 24-well plate and incubated at 37°C in 5% CO_2_ for 12 h. *S. aureus* was grown in BHI broth with or without Sal A to an OD_600_ of 1.0. Then, the bacterial suspension was added to the plate and incubated for 1 h. The bacterial suspension was discarded, and the A549 cells were washed three times with PBS and treated with DMEM containing 300 μg/ml gentamycin. After incubation for 2 h, the plates were washed three times, and the cells were lysed with sterile distilled water. The lysates were diluted 100-fold in PBS and spread onto BHI agar plates in triplicate. The plates were incubated at 37°C for 12 h, and the number of colony-forming unit (CFU) was determined by manual counting.

### Western blot

The dissolved cell wall proteins and cytoplasmic membranes were obtained according to a previously reported method [10]. Equal volumes of protein extract were separated using a 12% SDS-PAGE gel, and the proteins were transferred onto polyvinylidine difluoride membranes (Wako Pure Chemical Industries, Ltd., Osaka, Japan). After blotting, the membrane was blocked with 5% BSA for 2 h and probed with primary antibodies against *S. aureus* SrtA overnight at 4°C. Next, the membrane was washed and incubated with HRP-labelled goat anti-rabbit IgG. After washing again with PBS, the immunoreactive bands were visualized by an ECL Western blot detection system (GE Healthcare, UK). The primary antibodies against *S. aureus* SrtA were produced in-house by our team members. The secondary HRP-labelled goat anti-rabbit IgG antibody was obtained from Proteintech (Chicago, USA).

### Biolayer interferometry assay

The kinetics of the binding of Sal A to SrtA were determined by a biolayer interferometry (BLI) assay using the Octet RED96 system (ForteBio, Inc., Menlo Park, CA) as described previously [24]. Ni-NTA sensors were used to bind the His-tag SrtA protein. The assay buffer used was PBS containing 0.1% bovine serum albumin and 0.02% Tween-20, pH 6.5. Before the assay was performed, the Ni-NTA sensors were pre-wetted in assay buffer for 10 min, and then the Ni-NTA sensors were flushed with buffer for 60 s to generate the baseline. SrtA was immobilized on the surface of the sensor tip by incubating it in 1.5 mM protein solution for 3600 s. The excess protein was removed by washing the sensor with buffer for 300 s. Subsequently, the sensor was exposed to a cycle of 60 s for the baseline step, 200 s for the association step and 100 s for the dissociation step in different concentrations of Sal A solution (250, 125, 62.5, 31.25 and 15.625 μM). The binding of the sensor to protein without the loading of Sal A served as a control. The data were processed using the double reference subtraction method in Forte Bio analysis software (v6.4).

### Molecular docking and dynamic simulation

The molecular docking studies were performed using Autodock Vina 1.1.2 [25]. The 3D structure of SrtA (PDB ID: 1T2P) was obtained from the Protein Data Bank. The 3D structures of Sal A were built using the Hyperchem 8.0 software package (CambridgeSoft, MA, USA). The graphical user interface AutoDockTools 1.5.6 software package [26,27] was employed to generate the docking input files. For molecular docking, the default parameters were used for all docking calculations, unless otherwise stated. After molecular docking, the best docked pose obtained for the Sal A-SrtA complex was subjected to 40 ns molecular dynamics simulation, which were conducted using processes performed as described previously [28].

### Fluorescence quenching assay

The fluorescence quenching assay was performed as previously described [29]. Briefly, purified SrtA and mutant protein were diluted to 500 ng/ml with PBS. A total of 2.5 ml diluted protein solution was mixed with Sal A at different concentrations ranging from 0 to 4.5 μg. After incubation for 10 min, the fluorescence spectra of the mixed solutions were measured using a fluorescence spectrophotometer (RF5301, Japan). For the fluorescence measurement, the excitation and emission slits were set at 5.0 nm, the excitation wavelength was set at 280 nm, and fluorescence emission spectra in the range of 290–500 nm were scanned; the fluorescence intensity at 340 nm was recorded. All assays were performed in triplicate.

### Pneumonia model experiment

The animal experiments were performed using 6- to 8-week-old female C57BL/6J mice obtained from the Experimental Animal Center of Jilin University and conducted according to the guidelines of the Animal Care and Use Committee of Jilin University. The mouse *S. aureus* pneumonia model was generated as described previously [30]. Briefly, the mice were anaesthetized with ether and inoculated with 30 μl bacterial resuspension via the left nare. The mice were then held upright to allow the bacteria to be inhaled into the lungs. For the survival assessment, groups of 10 mice were challenged with 2 × 10^9^ CFU *S. aureus.* One hour after intranasal inoculation, the mice were subcutaneously injected with 100 mg/kg of Sal A, 75 mg/kg of latamoxef or 100 mg/kg of Sal A plus 75 mg/kg of latamoxef at 12 h intervals, and survival was assessed for 96 h. For the bacterial load, histopathological, and bronchoalveolar lavage fluid (BALF) experiments, mice were inoculated with 1 × 10^9^ CFU *S. aureus*. The mice were then given a subcutaneous injection of 100 mg/kg Sal A, 75 mg/kg latamoxef or a combination of 100 mg/kg Sal A and 75 mg/kg every 12 h. After 24 h, the left lung of each anaesthetized mouse was aseptically excised and homogenized, and the CFU was determined by serial dilutions. The right lungs from each group of mice were excised for histopathological analysis, and the samples were fixed in 10% formalin and stained with haematoxylin and eosin (H&E).

For the determinations of the levels of TNF-α, IL-6, IL-1β, 24 h after inoculation with bacteria, the lungs of anaesthetized mice were lavaged with a tracheal cannula according to a procedure described previously [31]. The lavage samples were centrifuged for 10 min at 500 × g and 4°C. The supernatant was collected, and the amounts of cytokines were quantified using commercial enzyme-linked immunosorbent assay (ELISA) kits (eBioscience, San Diego, CA) according to the manufacturer’s protocols. All animal experiments were replicated at least twice, with essentially identical results.

## Results

### Identification of Sal A as an inhibitor of SrtA

**Figure 1.**
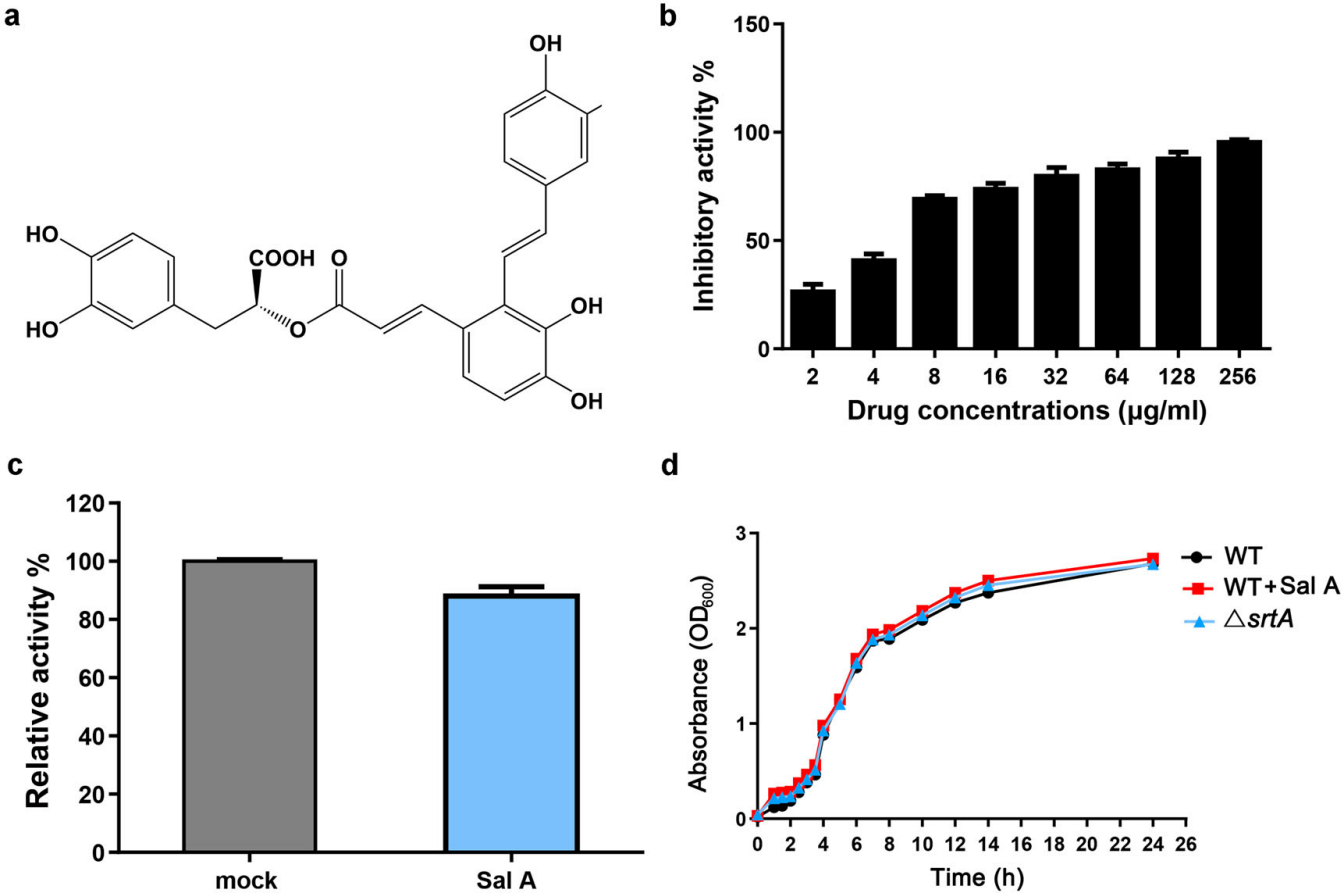
Sal A inhibits the activity of SrtA without affecting the growth of *S. aureus.* (a) The chemical structure of Sal A, (2R)-3-(3,4-Dihydroxyphenyl)-2-[(E)-3-[2-[(E)-2-(3,4-dihydroxyphenyl)ethenyl]-3,4-dihydroxyphenyl]prop-2-enoyl]oxypropanoic acid. (b) Inhibitory effect of Sal A on the activity of SrtA *in vitro*. (c) Reversible inhibition of SrtA by Sal A. SrtA was treated with buffer or with compound Sal A at a concentration of 10 × IC_50_ and diluted, and SrtA activity was determined as Abz-LPATG-Dap(Dnp)-NH_2_ cleavage. Mock-treated SrtA was assigned 100% activity. (d) The growth curves of the △*srtA* group and the *S. aureus* group treated with or without Sal A (256 μg/ml). In each panel, data from three independent experiments are presented.

The bioactive natural products in traditional Chinese herbs have become an important source of inhibitors against virulence factors such as *S. aureus* SrtA [32]. Fluorescence resonance energy transfer (FRET) have been widely used to screen for inhibitors of SrtA. In the present study, natural compounds from Chinese herbs were screened for SrtA inhibitor by FRET, Sal A (Figure 1(a)) was identified as an effective inhibitor of SrtA in *S. aureus*. As shown in Figure 1(b), Sal A inhibited the activity of SrtA in a concentration-dependent manner. The IC_50_ value was calculated to be 5.75 μg/ml. Because Sal A has high inhibitory activity and water solubility, we further analyzed whether the inhibition of SrtA by Sal A was reversible. Following the dilution of SrtA/Sal A to 10-fold the IC_50_ value, 88 ± 3.21% of SrtA activity was recovered in comparison with the control group (Figure 1(c)), indicating that Sal A is a reversible inhibitor of SrtA that non-covalently interacts with the active site of SrtA [33]. Unlike antibiotics, which rapidly kill bacteria, the addition of Sal A at a concentration of 40-fold the IC_50_ (up to 200 μM) to *S. aureus* cultures did not inhibit the growth of *S. aureus* (Figure 1(d)). Using a MIC assay, we measured the MIC of Sal A to be greater than 1,024 μg/ml, suggesting that Sal A inhibits SrtA without inhibiting the growth of *S. aureus*.

### Sal A inhibits the adhesion of *S. aureus* to fibrinogen

**Figure 2.**
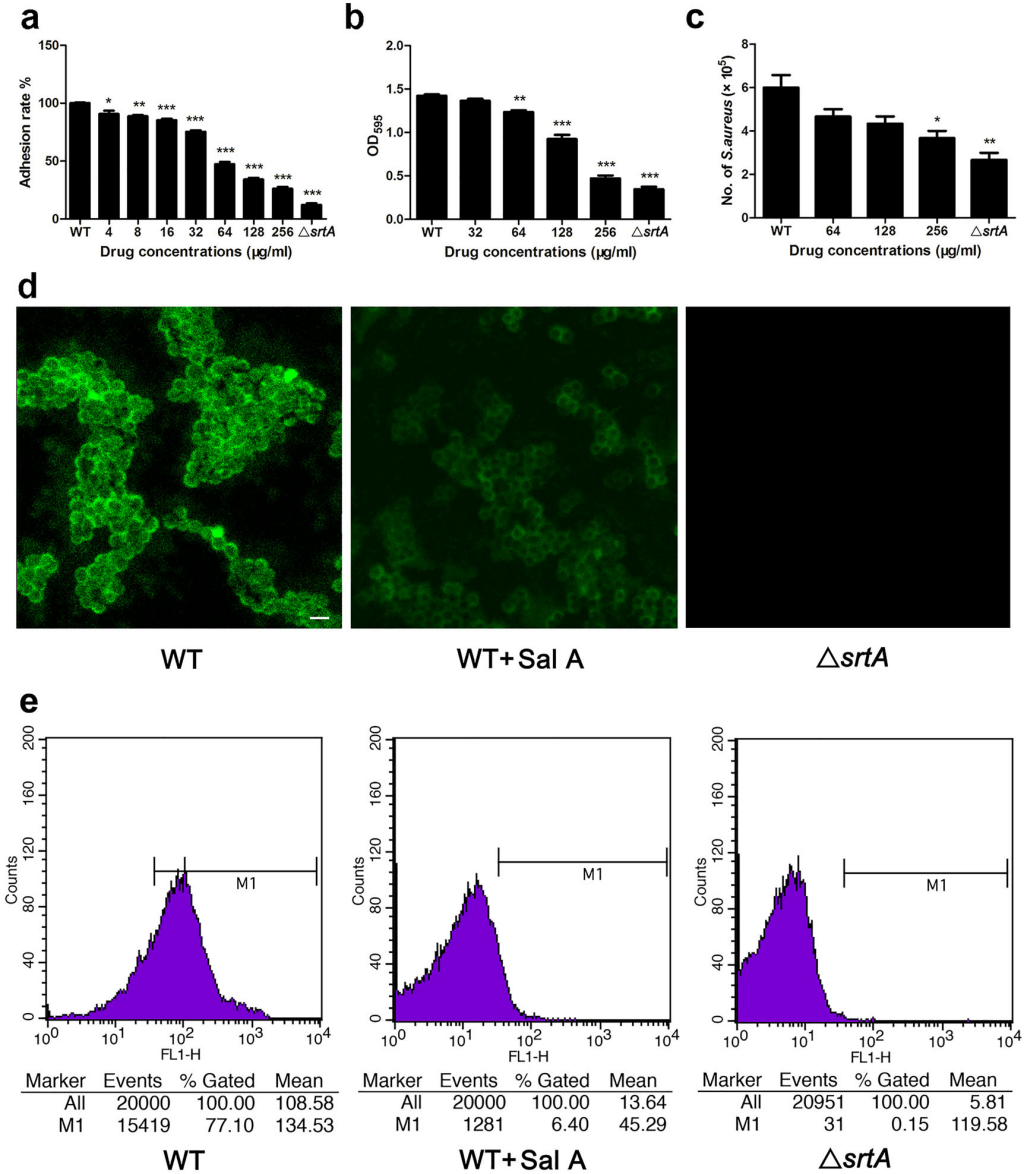
Sal A inhibits the adhesion of *S. aureus* to fibrinogen, the formation of biofilms, the anchoring of SpA to the bacterial surface and the invasion of *S. aureus* into A549 cells. (a) The effect of Sal A on the adhesion of *S. aureus* to fibrinogen. (b) Biofilm formation of *S. aureus* in the presence of Sal A. The formation of biofilms was quantified by crystal violet (CV)-staining biofilm assays. (c) The effect of Sal A on the internalization of *S. aureus* into A549 cells. The number of bacteria in the cells was quantified by serial dilution on LB agar plates after A549 cell lysis. (d) Fluorescence micrographs of *S. aureus* stained with FITC-labeled rabbit IgG. Confocal laser-scanning microscopy was utilized to observe the binding of FITC-labelled IgG to SpA. The intensity of the green fluorescence indicated the amount of SpA anchored to the surface of *S. aureus*. Scale bar, 1 µm. (e) SpA on the surface of *S. aureus* was detected using FITC-conjugated rabbit IgG, and the fluorescence intensity was quantified using flow cytometry. In each panel, the Δ*srtA* group serves as the positive control. Three independent experiments were performed to obtain stable results. The results are expressed as the mean ± SEM. *P < 0.05, **P < 0.01 and ***P < 0.001 compared with the WT group.

In *S. aureus,* many adhesins involved in adherence to the host cell, such as ClfA/ClfB and fibronectin binding proteins (FnBPs) [34], are anchored by SrtA. To verify whether Sal A can reduce the adhesion of *S. aureus* to fibrinogen by inhibiting SrtA, the extent of bacterial adhesion to fibrinogen was determined. As shown in Figure 2(a), Sal A inhibited the adhesion of *S. aureus* to fibrinogen in a dose-dependent manner. The Δ*srtA* group showed the lowest adhesion rate, which was only 12 ± 1.53%. These results indicated that the inhibition of SrtA by Sal A reduced the adhesion of bacteria to fibrinogen.

### Sal A inhibits the biofilm formation of *S. aureus*

The primary stage of biofilm formation is bacterial adhesion, which is mediated by cell surface proteins that are anchored by SrtA [35], Therefore, inhibition of SrtA should cause reduced anchor of various cell surface proteins that affect biofilm formation. Since sal A inhibits SrtA *in vitro*, we further tested Sal A using a *S. aureus* biofilm assay. The *S. aureus* cells were cultured in BHI broth supplemented with different concentrations of Sal A and the biofilm biomass was quantified. The Δ*srtA* group served as a positive control. As shown in Figure 2(b), we observed that Sal A reduced biofilm formation in a dose-dependent manner, and the inhibition by 256 μg/ml Sal A of biofilm formation was 33.14% compared with the untreated group, which is similar to that observed in the Δ*srtA* group (24.26%), indicating that Sal A can reduce *S. aureus* biofilm formation by inhibiting SrtA activity.

### Sal A inhibits the invasion of *S. aureus* into A549 cells

As many bacterial surface proteins anchored by SrtA function in the adhesion and invasion of host cells, we further investigated the effect of SrtA inhibition on the internalization of *S. aureus* into A549 cells. As shown in Figure 2(c), both the *S. aureus* group treated with Sal A and the Δ*srtA* group showed a significant reduction in the number of bacteria entering the cells compared to the control group, indicating that treatment with Sal A attenuated the invasion of *S. aureus* into A549 cells by inhibiting the anchoring of bacterial surface proteins.

### Sal A inhibits the anchoring of surface protein A in *S. aureus*

Next, we analyzed the amount of SpA in *S. aureus* treated with Sal A by performing the fluorescence staining. The results showed that the Δ*srtA* group showed no green fluorescence when compared with the WT group (Figure 2(d)). After treatment with 256 μg/ml Sal A, the level of green fluorescence was considerably reduced compared with the WT group. The level of SpA anchored on the bacterial surface was further quantified using flow cytometry. As shown in Figure 2(e), the mean value of fluorescence yield in the Δ*srtA* group was only 5.81, indicating that no SpA is anchored on the bacterial surface when *srtA* is not expressed. However, the mean values of fluorescence yield in the Sal A-treated group significantly decreased compared with the untreated WT *S. aureus* (13.64 versus 108.58), indicating that Sal A reduced the level of SpA displayed on the surface of *S. aureus*. Taken together, these results demonstrated that Sal A can interfere with the assembly of SpA in the cell wall by inhibiting the activity of SrtA.

### Sal A has no effect on the expression of SrtA

**Figure 3.**
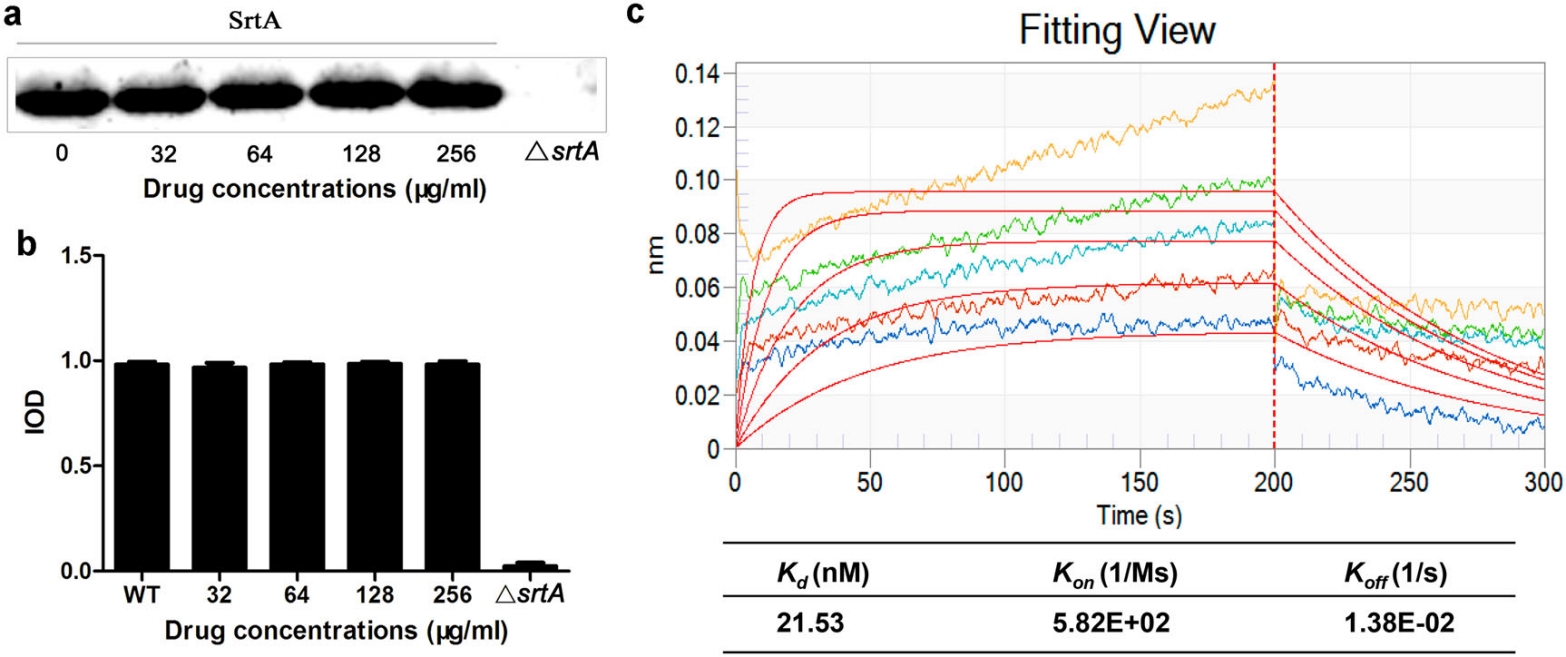
The expression of SrtA in the presence of Sal A and the binding kinetics of Sal A with SrtA. (a) Western blot analysis of SrtA. (b) Grayscale analysis of the SrtA protein bands. (c) Biolayer interferometry assay of the binding kinetics of different concentrations of Sal A with SrtA. The insets show the steady state curve fitting. The determined kinetic constants are given in the table.

To test whether Sal A affects the expression of SrtA, *S. aureus* was incubated with 256, 128, 64, or 32 μg/ml of Sal A, and the expression level of SrtA was detected by Western blotting. The results were further analyzed by grayscale analysis. The results showed that the expression level of SrtA in the Sal A treatment group was similar to that in the untreated group (Figure 3(a and b), indicating that Sal A did not impact the expression of SrtA.

### Determination of the molecular interaction between Sal A and SrtA

Next, we analyzed the interaction between Sal A and SrtA by performing a BLI assay. The sensorgrams showing the association and dissociation of different concentrations of Sal A with SrtA showed that Sal A exhibited slow association and dissociation with SrtA, and the binding of Sal A to the SrtA immobilized on the probe was concentration-dependent (Figure 3(c)). The *K_d_* value of Sal A in the presence of SrtA was 21.53 nM. These data indicate that Sal A binds to SrtA.

### Binding mode of Sal A with SrtA

**Figure 4.**
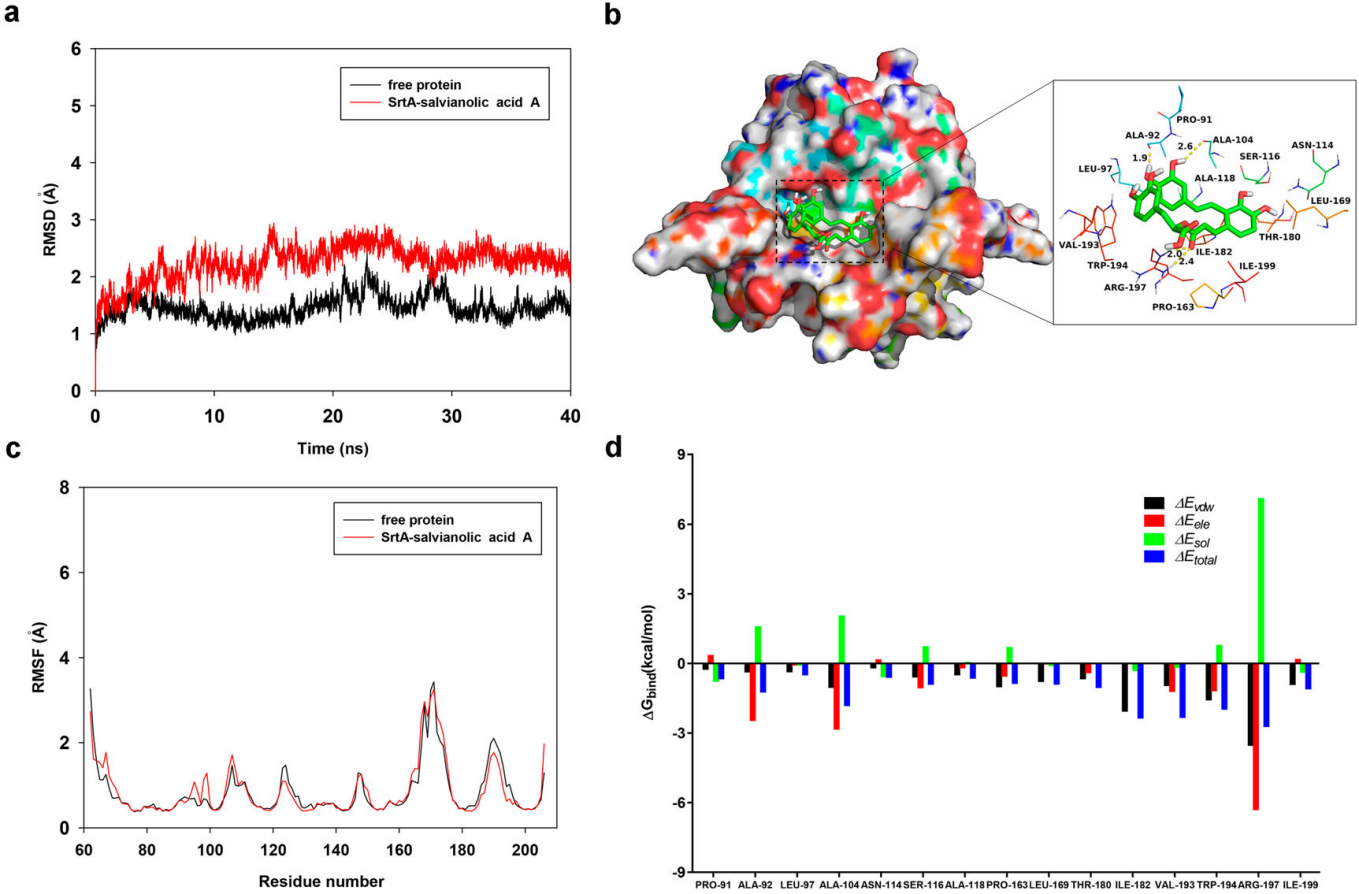
Determination of the binding mechanism of SrtA with the molecular modelling studies. (a) The root-mean-square deviations (RMSDs) of all the atoms in the SrtA-Sal A complex with respect to the initial structure as a function of time. (b) The predicted binding mode of Sal A in the SrtA binding pocket determined with MD simulations. (c) RMSFs of the residues of the whole protein in the SrtA-Sal A complex and free SrtA during the 40 ns simulation. (d) Decomposition of the binding energy on a per-residue basis in the SrtA-Sal A complex.

To elucidate the structural basis of the inhibitory effect of Sal A on SrtA, we performed 40-ns molecular dynamics simulation studies. As shown in Figure 4(a), the root-mean-square deviation (RMSD) values of the protein backbone during the simulation showed that the protein structures of the two systems were stabilized during the simulation. The root mean square fluctuations (RMSFs) of these residues are shown in Figure 4(c), all of the residues that bound to Sal A showed a small degree of flexibility, with RMSF values of less than 3.5 Å when compared to those of free SrtA, indicating that these residues are more rigid because of binding to Sal A. The summations of the per residue interaction free energies were separated according to the Van der Waals (Δ*E_vdw_*), electrostatic solvation (Δ*E_sol_*) and total contribution (Δ*E_total_*) and were calculated with the MMGBSA method. The calculation results revealed that Δ*E_ele,_* Δ*E_vdw_* and the hydrogen bond interactions were the major contributors to the binding of Sal A to SrtA. Residues Arg-197 and Ile-182, with the Δ*E_vdw_* of < – 2.0 kcal/mol, have strong Van der Waals interaction with the Sal A because of the close proximity between the residues and the Sal A. The Arg-197 residue formed electrostatic interactions with the carboxyl group of Sal A with a Δ*E_ele_* of < – 6.0 kcal/mol. The Ala-92 and Ala-104 residues made moderate electrostatic (Δ*E_ele_*) contributions with a Δ*E_ele_* of < – 2.0 kcal/mol (Figure 4(d)). In addition, the Arg-197 residue formed two strong hydrogen bond interactions with the carbonyl “O” of Sal A with bond lengths of 2.0 and 2.4 Å (Figure 4(d)). The Ala-92 and Ala-104 residues oriented the two hydroxyl groups of Sal A by forming two strong hydrogen bond interactions (bond lengths: 1.9 and 2.6 Å) (Figure 4(b)). Moreover, Δ*E_total_* for the SrtA-Sal A complex was calculated, and the Δ*G_bind_* of Sal A was 25.0 kcal/mol, suggesting that Sal A binds tightly to the substrate binding pocket of SrtA.

To verify the predictions of the molecular dynamics simulation, four SrtA mutants, A104L-SrtA, A92L-SrtA, R197A-SrtA, and I182A-SrtA, were constructed and expressed. The binding constants (*K_A_*) between Sal A and WT SrtA and its mutants were determined by a fluorescence quenching assay according to a method described previously [21]. The binding constants *K_A_* between Sal A and SrtA and its mutants are shown in Table 2. They showed that WT SrtA and Sal A had *K_A_* values of 5.8 × 10^4 ^l/mol. However, for A104L-SrtA, A92L-SrtA, R197A-SrtA, and I182A-SrtA, *K_A_* was sharply decreased, indicating that Ala-104, Ala-92, Arg-197, and Ile-182 of SrtA are necessary for the binding of Sal A to SrtA. These results are consistent with the predictions of the molecular dynamics simulation, suggesting that the information generated by the molecular modelling was reliable.

**Table 2.** The binding constant (K_A_) values based on fluorescence spectroscopy quenching.

Proteins	WT-SrtA	A92L	A104L	I182A	R197A
***K_A_* (1 × 10^4^) l/mol**	5.8	3.78	2.62	1.03	2.22
**n**	0.9013	0.983	0.9055	0.8357	0.9064

### The combination of Sal A with latamoxef protects mice from *S. aureus*-induced lethal pneumonia

**Figure 5.**
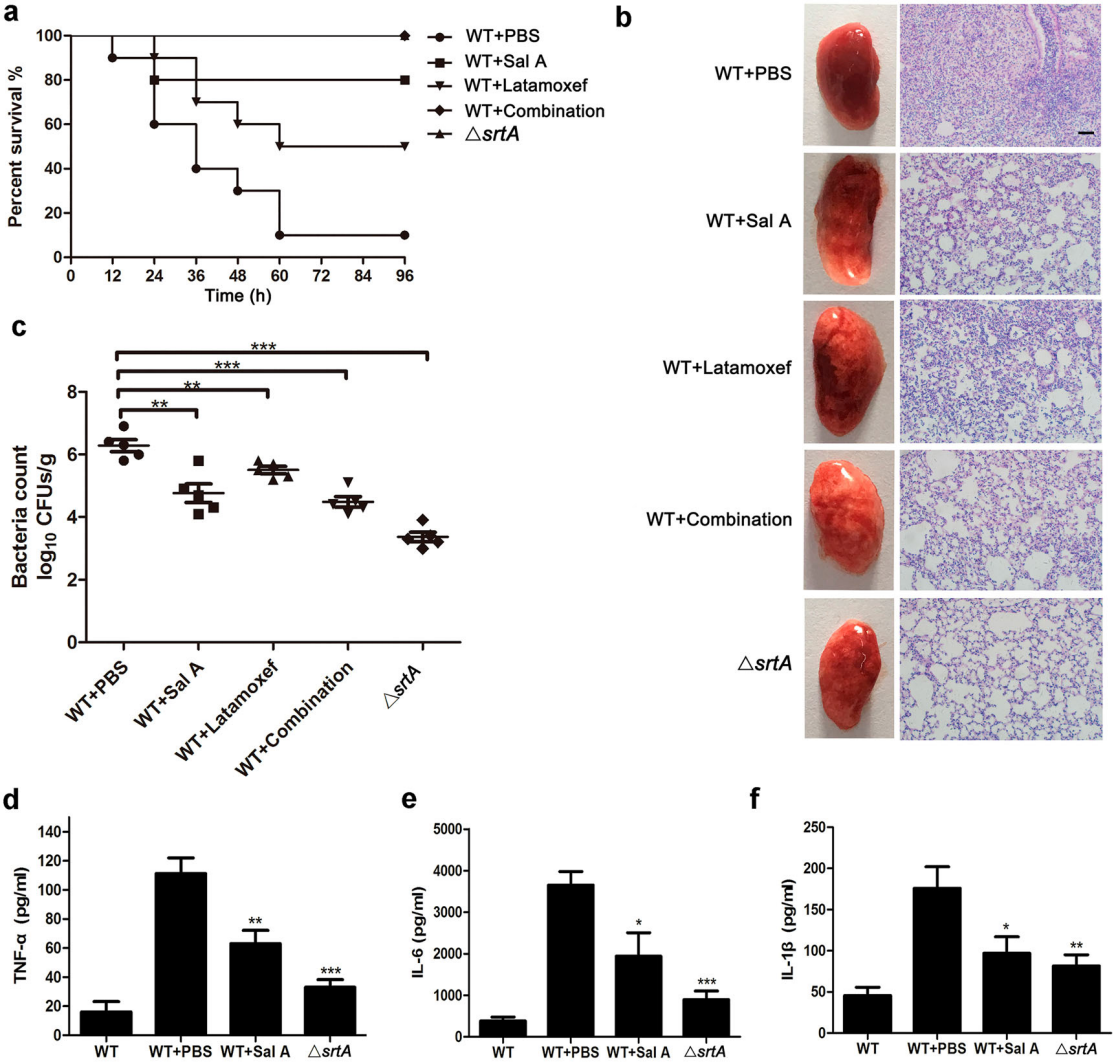
Sal A protects mice from *S. aureus*-induced lethal pneumonia. (a) The effect of Sal A on the survival rate in mice challenged with 2 × 10^9 ^CFU of *S. aureus* USA300 or the △*srtA* strain (10 mice/group) via the i.n. route. The mice were treated with Sal A and/or latamoxef (1 h postinfection, twice a day). The survival of mice was recorded at 12 h intervals for 96 h. (b) Overall pathological changes and histopathological changes in lung tissue from mice treated with or without Sal A. Mice were challenged with 1 × 10^9^ CFU of *S. aureus* USA300 or the △*srtA* strain and were treated with or without Sal A and/or latamoxef (1 h post infection, twice a day). At 24 h postinfection, the lungs were removed, and the gross pathological changes and the histopathological changes in the lungs were examined. Scale bar, 50 μm. (c) The bacterial load in the lungs 24 h after infection. Mice were challenged with 1 × 10^9^ CFU of *S. aureus* via the i.n. route and then were treated with or without Sal A and/or latamoxef (1 h postinfection, twice a day). At 24 h postinfection, the lungs were removed, and the bacterial burdens in the lungs were quantified. (d-f) Levels of TNF-α, IL-6 and IL-1β in the bronchoalveolar lavage fluid (BALF) of mice challenged with 1 × 10^9^ CFU of *S. aureus* USA300. The mice were treated with Sal A or/and latamoxef (1 h postinfection, twice a day). The cytokine levels in BALF were determined 24 h after infection. In each panel, the data shown were collected from three independent experiments and are presented as the mean ± SEM; *P < 0.05, **P < 0.01 and ***P < 0.001 using a 2-tailed *t* test.

As Sal A exhibits inhibitory effects on SrtA and reduces the invasion of *S. aureus* into A549 cells, we further studied whether Sal A has good therapeutic effects during *S. aureus* infection in a mouse model. The mice were infected with 2 × 10^9^ CFU of *S. aureus* USA300 or the *srtA* mutant strain. Mice treated with PBS after infection served as negative controls. Sal A, latamoxef or a combination of Sal A and latamoxef was administered to determine the protective effect on mice challenged with *S. aureus*. As shown in Figure 5(a) the survival rate of mice infected with *S. aureus* treated with PBS was only 10% compared with that of mice infected with *S. aureus* Δ*srtA*. When treated with 100 mg/kg Sal A or 75 mg/kg latamoxef for 4 d, the survival rate was increased to 80% for Sal A and 50% for latamoxef. When Sal A and latamoxef were combined, the survival rate increased to 100% compared with the control group (WT + PBS), demonstrating a significant survival advantage and indicating that the combination of Sal A and latamoxef showed better protective efficacy than monotherapy.

### The combination of Sal A with latamoxef alleviated injury and reduced bacterial loads in lung tissue

To further assess the protective effects of different treatments on lung tissues, groups of C57BL/6J mice were challenged intranasally (i.n.) with a sublethal dose of 1 × 10^9^ CFU of *S. aureus*, *S. aureus* Δ*srtA* or PBS and were then subcutaneously injected with 100 mg/kg of Sal A, 75 mg/kg of latamoxef or a combination of Sal A with latamoxef. The mice were euthanized 24 h after treatment, and histopathological analysis of the lungs was performed to evaluate the effect of Sal A monotherapy and combination therapy. As shown in Figure 5(b), the lung tissue from the control group was red and hard, while the lungs from the Δ*srtA*-infected group, monotherapy and the combination therapy groups were pink and spongy. Histopathological examination showed that many inflammatory cells had accumulated in the alveolar sulcus, and conspicuous consolidation was observed in the lungs of the control group. The lungs of the Δ*srtA* group had no obvious histopathological changes other than an incomplete alveolar structure, which was consistent with previous studies [36]. After treatment with Sal A, latamoxef or combination of Sal A with latamoxef, lung tissue congestion was reduced compared with that in the control group, the infiltration of inflammatory cells in the alveolar cavity was significantly reduced, and the alveolar structure was relatively complete, suggesting that pulmonary inflammation had been alleviated. The effect of different treatments on the bacterial load in the lungs was further examined. As shown in Figure 5(c), treatment with Sal A or latamoxef or a combination of Sal A and latamoxef significantly decreased the viable MRSA loads in comparison with those in the control. Furthermore, the levels of TNF-α, IL-6, and IL-1β in the BALF of mice were quantified. Compared with the control group, Sal A significantly downregulated the levels of inflammatory cytokines in the BALF of mice (Figure 5(d–f)).

## Discussion

The emergence of MRSA indicates an urgent need for the control of the use of antibiotics and the development of novel therapeutic agents [37]. *S. aureus* SrtA has been considered a promising target of anti-infective therapy. Here, we revealed that Sal A is an effective SrtA inhibitor. In combination with antibiotics, it offers full protection against MRSA-induced lethal pneumonia. Sal A is a major bioactive component of the commonly used traditional Chinese medicine *Salvia miltiorrhiza,* which is extensively used in the treatment of cardiovascular diseases. The growth curve of *S. aureus* treated with Sal A indicated that Sal A did not have any antibacterial effects against *S. aureus* (Figure 1(d)). This means that it exerts little to no selective stress on *S. aureus* and thus its clinical application will not induce the occurrence of drug resistance and affect normal microbiota in the body.

Further experiments revealed that the inhibition of the activity of SrtA by Sal A reduced the adherence of *S. aureus* USA300 to fibronectin (Figure 2(a)), the formation of biofilms (Figure 2(b)), the display of SpA on the cell wall (Figure 2(d and e)) and the internalization of *S. aureus* USA300 into A549 cells (Figure 2(c)). The decreased internalization of *S. aureus* after treatment with Sal A (Figure 1(c)) may be due to reduced adhesion caused by the inhibition of SrtA. We further investigated the molecular mechanism by which Sal A inhibits SrtA activity by molecular dynamics simulation. The results of the molecular dynamics simulation and fluorescence quenching experiments revealed that the SrtA residues Ala-92, Arg-197, I182, and Ala-104 play a major role in the interaction between Sal A and SrtA (Figure 4(c and d), Table 2).

Targeting SrtA is an effective approach to attenuate the pathogenesis of *S. aureu*s to facilitate clearance by the host immune system. We observed that Sal A has potent therapeutic effects against *S. aureus* infections *in vivo*, and treatment with Sal A significantly weakened the virulence of *S. aureus* and increased the survival rate of infected mice. When mice were treated with Sal A in combination with latamoxef, all mice challenged with the lethal dose of MRSA strain USA300 survived, which demonstrated the strong curative effect of Sal A. In addition, the *S. aureus* loads in the lungs of mice treated with Sal A combined with latamoxef were significantly reduced compared to those in untreated mice. This result may be attributed to the decreased adhesion and internalization of *S. aureu*s by epithelial cells caused by the inhibition of SrtA.

Acute pneumonia is usually accompanied by the excessive activation of the inflammatory response, which is manifested by the accumulation of inflammatory cells and leads to severe tissue damage [38]. As Sal A has been reported to possess strong anti-inflammatory activity [39], we further investigated the levels of the proinflammatory cytokines TNF-α, IL-6 and IL-1β in BALF. The result of the ELISA showed that Sal A can downregulate the expression levels of the proinflammatory cytokines TNF-α, IL-6, IL-1β in mice treated with Sal A. The gross pathological changes and histopathological examination also indicated that treatment with Sal A could inhibit the progression of inflammation and alleviate injury in lung tissues.

Overall, our results demonstrate that Sal A is an effective inhibitor of SrtA. Treatment with Sal A conferred protection against lethal pneumonia in rats through the inhibition of the virulence of *S. aureus* and the inflammation of the host. As Sal A has good water solubility and is a commonly used drug for the treatment of myocardial ischaemic diseases in the clinic, it may be a safe and promising adjuvant for conventional antibiotic therapy to combat antibiotic-resistant *S. aureus* infections.

## Supplementary Material

Supplemental Material
