# Supplementary figures and images for: The combination of salvianolic acid A with latamoxef completely protects mice against lethal pneumonia caused by methicillin-resistant Staphylococcus aureus
Source: Emerg Microbes Infect. 2020 Jan 23;9(1):169–79. doi: 10.1080/22221751.2020.1711817 (PMC7006784; doi:10.1080/22221751.2020.1711817)

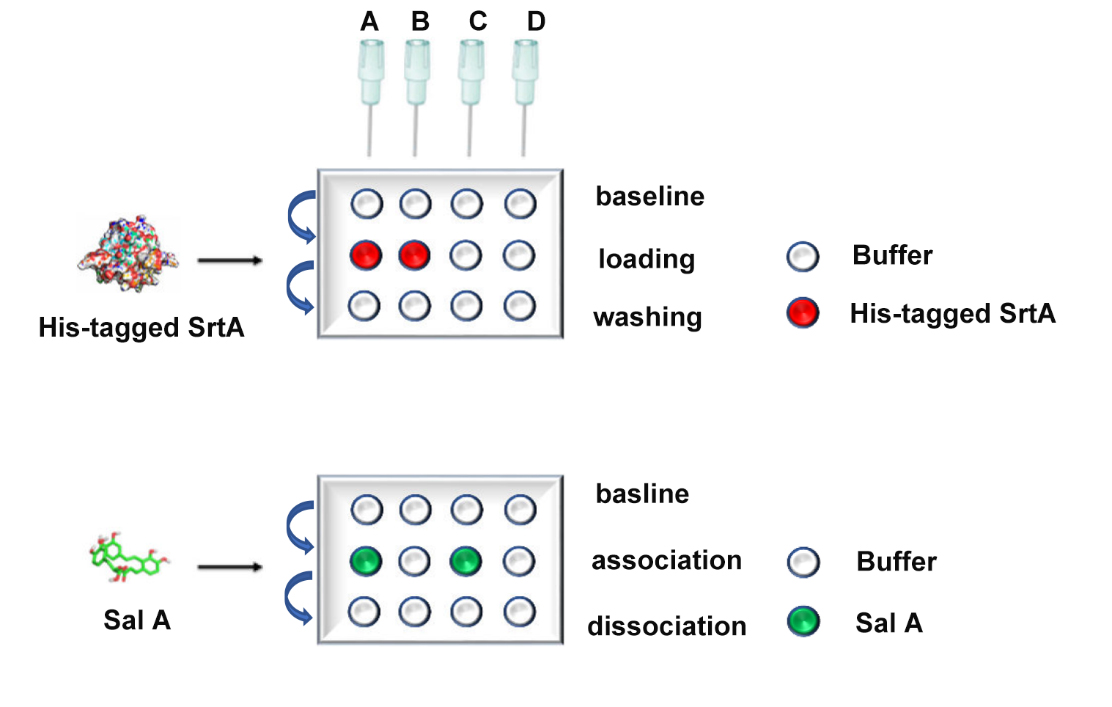

Supplement: Supplemental Material [file TEMI_A_1711817_SM8239.jpg]
